# Multisystemic Side Effects of an Indispensable Old Drug: A Case Report of Chronic Lithium Use (A Patient with Multiple Side Effects of Lithium)

**DOI:** 10.1155/2015/473931

**Published:** 2015-10-28

**Authors:** Levent Demirtas, Emin Murat Akbas, Husnu Degirmenci, Ali Gurel, Eren Duzgun

**Affiliations:** ^1^Department of Internal Medicine, School of Medicine, Erzincan University, 24100 Erzincan, Turkey; ^2^Department of Endocrinology, School of Medicine, Erzincan University, 24100 Erzincan, Turkey; ^3^Department of Cardiology, School of Medicine, Erzincan University, 24100 Erzincan, Turkey; ^4^Mengucek Gazi Training and Research Hospital, Department of Nephrology, 24100 Erzincan, Turkey

## Abstract

Presented here is a case of long-term lithium use, with multiple emerging lithium-associated side effects. An 82-year-old woman was brought into the emergency department because of loss of consciousness. According to the physical examination and laboratory analyses, patient was diagnosed with lithium-associated hypercalcemia, hyperparathyroidism, nephrogenic diabetes insipidus (NDI), symptomatic sinus bradycardia, and thyroid dysfunction. In the literature, there is a limited number of case reports with lithium induced multiple clinical conditions. Multiple clinical manifestations due to the side effects of chronic lithium use might be seen. Health care professionals should keep in mind that lithium-related side effects might trigger or exacerbate each other. To avoid toxicity, close follow-up and clinical supervision are important for the early diagnosis and treatment of these side effects, due to the narrow therapeutic index and obscure clinical signs and symptoms of toxicity.

## 1. Introduction

Lithium is one of the first treatment options for bipolar affective disorder and it has been used in modern psychiatric treatments since 1949 [1]. Although its efficacy has been proven as a prophylactic in the relapse and recurrence of unipolar depression, hypomania, mania, short-term mortality, and suicidal risk, it has many side effects [1, 2].

The clinical problems caused by lithium include narrow therapeutic index, cardiac toxicity, thyroid abnormalities, hyperparathyroidism, transient hyperglycemia, and nephrogenic diabetes insipidus (NDI) caused by renal tubular dysfunction [1, 3, 4]. Multiple side effects from lithium are rarely seen in the same patient. Therefore, we present a case study of long-term lithium use in a patient diagnosed with lithium-associated hypercalcemia, hyperparathyroidism, NDI, symptomatic bradycardia, and thyroid dysfunction. Our aim was to discuss the combination of multiple side effects because the symptoms of lithium intoxication are often overlooked by physicians. Moreover, we discuss the finding that the problems caused by lithium may be associated with each other and aggravate each other.

## 2. Clinical Presentation and Intervention

**Figure 1:**
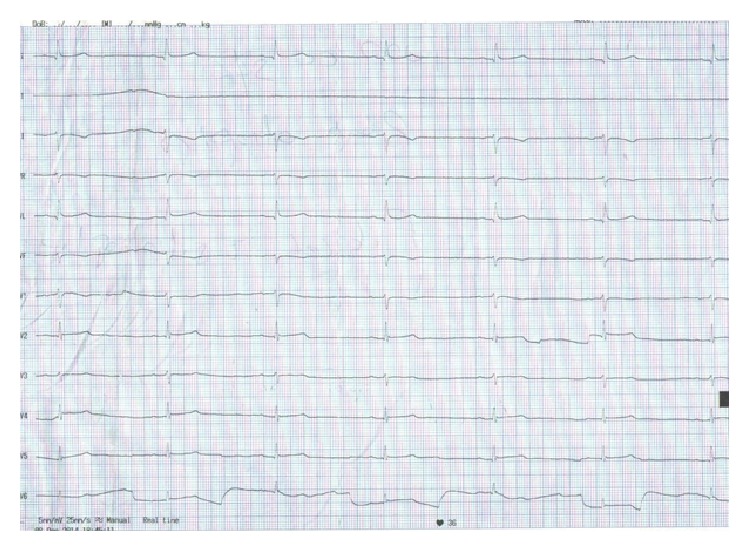
Sinus bradycardia without signs of ischemia on ECG.

An 82-year-old woman was brought to the emergency department with mental confusion, and it was determined that she had complaints of weakness, fatigue, excessive thirst with increasing severity, frequent urination, and palpitations over the previous year. Her medication history was as follows: lithium, 300 mg twice daily; quetiapine, 25 mg once daily for a bipolar mood disorder for 20 years; ramipril, 2.5 mg daily for hypertension for 10 years; levodopa/benserazide, 100/25 mg three times daily for Parkinson's disease for two years; ibandronic acid, 150 mg monthly; and calcium/cholecalciferol, 1000/880 mg daily for osteoporosis for one year. There was no history of nonsteroidal anti-inflammatory drugs (NSAIDs), thiazide, and loop diuretics use. The patient had not previously been under our follow-up care and her relatives did not declare a history of lithium intoxication. The patient's lithium levels and renal functions were within normal limits during regular monitoring within the previous one-year period. Two days before hospitalization, a psychiatrist discontinued the lithium treatment because the patient's blood lithium level was 1.3 mmol/L (0.6–1.2). The physical examination findings were as follows: 36 beats/min heart rate via electrocardiogram (ECG) with sinus bradycardia, without signs of ischemia on ECG (Figure 1), blood pressure 80/50 mmHg, and body temperature 36.8°C. Additionally, weak cooperation and orientation, decreased skin turgor tone, dry skin, and mucous membranes were detected. Upon admission, the patient's laboratory values were reported as follows: glucose 8.82 mmol, BUN 10.71 mmol/L (2.9–8.2), creatinine 114.92 *μ*mol/L (53–106), albumin 39 g/L (35–50), sodium 134 mmol/L (135–145), potassium 4.7 mmol/L (3.5–5.2), calcium 2.9 mmol/L (2.05–2.55), thyrotropin 0.145 mIU/L (0.4–4.2), free thyroxine 10.55 pmol/L (12–30), triiodothyronine 3.45 pmol/L (5.08–7.39), cortisol 496.86 nmol/L (140–690), and PTH 128 ng/L (10–65). The values observed in the blood gas analysis were as follows: pH 7.31 (7.35–7.45), PCO_2_ 4.68 kPa (4.7–5.9), and HCO_3_ 17.2 mmol/L (21–28). Based on the urinalysis, the urine density was 1.000, while the calculated plasma osmolality was 297 mmol/kg (275–295).

The patient was hospitalized in the cardiology clinic with sinus bradycardia due to lithium toxicity, and a temporary pacemaker was inserted. Her central venous pressure (CVP) was measured as 6 cm H_2_O. Intravenous hydration treatment was begun, based on the CVP and urine output. On the 3rd day of hospitalization, the transient bradycardia was improved and the pacemaker was removed. Because of the increased sodium level (162 mmol/L), the patient was transferred to the internal medicine clinic with a diagnosis of lithium-induced NDI.

At the internal medicine clinic, the intravenous hydration therapy was revised based on the plasma sodium level and urine output because the CVP value had decreased to 2 cm H_2_O and the plasma osmolality had increased to 341 mmol/kg. On the 5th day of hospitalization, the patient's blood lithium level was 0.02 mmol/L. During the follow-up period, despite hydration, the sodium levels did not decrease significantly, and the daily urine output was still 6–8 L. The water deprivation test could not be performed in our patient due to severe dehydration; therefore, desmopressin acetate (trihydrate), at 120 mcg twice daily, was begun with a presumptive diagnosis of lithium-induced NDI. On the 2nd day, the desmopressin acetate (trihydrate) dose was increased to 120 mcg three times daily, because the urine output was still 6 L/day, despite the fact that the sodium level had decreased to 156 mmol/L, and the patient's consciousness had partially improved. On the 6th day of the desmopressin treatment, the urine output decreased to 2.5 L/day, the sodium value decreased to 140 mmol/L, and the plasma osmolality decreased to 297 mmol/kg. Additionally, the urine density was 1.008, and the calcium levels reverted to normal levels. Both the consciousness and oral intake were completely improved, and the patient was discharged. On the 7th day after discharge, her daily urine output was 2.5–3.5 L, and no electrolyte imbalance was detected.

## 3. Discussion

Lithium salts are effective and inexpensive agents that have been used in the treatment of bipolar disorders for a long time [1]. Although the drug is cost effective, many side effects limit its use. The side effects associated with lithium are generally dose related [5]; therefore, the lowest effective dose should be used. Possible side effects include fine tremor of the hands, nystagmus, nausea, diarrhea, headache, weight gain, hypercholesterolemia, thyroid dysfunction, hypercalcemia/hyperparathyroidism, NDI, and renal injury [5]. In addition, the chronic use of lithium or acute high doses of the drug may cause neurological effects, such as coarse tremor, confusion, lethargy, seizures, coma, and cardiac, ophthalmological, and dermatological effects [5]. Close follow-up and clinical supervision are important for the early diagnosis and treatment of these side effects, due to the narrow therapeutic index and obscure clinical signs and symptoms of lithium toxicity.

Our patient was admitted with lithium-associated complaints, including symptomatic sinus bradycardia, hypercalcemia, hyperparathyroidism, NDI, and thyroid dysfunction, although she stopped using lithium a short time before her admission. A lithium level at the time of admission may be more helpful for obtaining a clear clinical picture. However, we also think that this patient's condition was due to chronic exposure more than to acute toxicity. No additional stress factors could be determined (infection, ischemic heart disease, cardiovascular event (CVE), etc.). Upon the evaluation of the patient, our opinion was that the presence of chronic DI was relatively compensated by thirst and oral fluid intake; however, hypovolemia, resulting from the loss of consciousness due to cardiac problems, made the symptoms obvious. All of these side effects could have been prevented if standard treatment would have been administered and follow-up had been provided. However, in this patient, possible symptoms of chronic DI may have been overlooked. The problems caused by lithium may aggravate each other, such as aggravated bradycardia, NDI, and dehydration by hypercalcemia and vice versa; and all of these problems may cause euthyroid sick syndrome (ESS). Thus, the clinical assessment might become more complicated. Supporting these complicated pathophysiological occurrence mechanisms, an appropriate treatment modality, such as rehydration, may resolve the complicated clinical issue more than expected. We think that, in the present case, an undetectable stress factor might have started a vicious circle and caused the clinical signs and symptoms manifested in this patient.

Cardiac effects associated with the use of lithium are observed in 20–30% of patients [6, 7]. These include chronic T-wave and ST-segment depression, conduction abnormalities, arrhythmias, and sinoatrial (SA) and atrioventricular (AV) node abnormalities that may lead to sinus bradycardia and SA blockage [5]. Among patients taking lithium, sinoatrial pathology generally occurs in the elderly population, and it usually manifests with syncope [8]. Although ECG changes are relatively common, serious cardiac toxicity signs are rare. Serious toxicity is usually related to advanced age, renal disease, existing cardiac disease, the use of nephrotoxic agents, and the agent's actions on the AV node [5, 6]. In this case study, no findings that caused sinus bradycardia, such as myocardial infarction or ischemia, were determined. There was not a drug use history associated with sinus bradycardia, other than lithium. There was no electrolyte imbalance, except hypercalcemia, and increased intracranial pressure and other etiologic pathologies, such as hypothermia, could not be determined. Hypercalcemia was associated with the patient's chronic lithium use. In the present case, after a cardiologist excluded other reasons for sinus bradycardia, lithium exposure was considered to be the reason for the sinus bradycardia with associated problems, such as thyroid dysfunction, hypercalcemia, and NDI.

In the chronic use of lithium, due to the increase in the set point of the calcium sensing receptor, hyperparathyroidism is observed with an absolute risk of 10% (versus 0.1% of the general population) [3, 4]. Additionally, hyperparathyroidism increases the renal tubular absorption of calcium, contributing to hypercalcemia. Lithium-associated hyperparathyroidism is frequently observed four times more often in women than in men. With the discontinuation of lithium treatment, hyperparathyroidism usually resolves spontaneously, but it can also continue to be high. In this condition, possible adenoma or hyperplasia may be the causative factors, and parathyroid surgery or medication with calcimimetic agents may be required [3].

NDI is defined as the inability of the kidneys to concentrate urine due to renal dysfunction, although normal levels of the antidiuretic hormone exist. Acquired NDI is diagnosed using a water deprivation test, and the urine osmolality of these patients is less than 300 mOsm/kg H_2_O, despite water deprivation and vasopressin treatment. Generally, an aquaporin-2 distribution problem caused by the G protein-coupled pathway disorder underscores the molecular basis of acquired DI [4].

Lithium causes NDI by accumulating in the distal tubular cells at a concentration that is 10–20 times higher than the serum concentration, leading to degenerative changes, necrosis, and a reduction in the aquaporin expression in the renal collecting ducts [9]. Lithium is the most common cause of acquired NDI and, in chronic lithium use, NDI can be observed in 20–40% of patients [3, 9, 10]. Although lithium may cause a decline in the glomerular filtration rate (GFR), in most patients it does so without clinical significance. Renal functions generally improve after the discontinuation of the drug; however, in some cases, it may take months or years to regain renal functions [3]. In addition, long-term use of lithium may cause irreversible injury due to interstitial nephropathy [3, 10]. Treatment consists of either addressing the causal disease or discontinuing the drug. Other probable useful treatment options are thiazide diuretics, NSAIDs, and low solute intake in food [11, 12]. Thiazide diuretics and NSAIDs have the potential to increase lithium toxicity and deterioration of renal function, so these drugs should be used with caution in these cases. Amiloride is the preferred diuretic, because, in addition to natriuresis, it prevents the entry of lithium into the tubular cells [11]; additionally, high doses of desmopressin have been observed to be effective in some NDI cases [11]. Our patient responded to high doses of desmopressin; however, the hypercalcemia detected in our case might be another reason for the acquired NDI, and the treatment for this high calcium level might have contributed to her recovery from NDI.

Thyroid dysfunctions may be observed in patients taking lithium medication, and goiter is the most common thyroid-associated clinical disorder. The prevalence of lithium-associated goiter differs according to geographic region, duration of lithium use, and diagnostic methods [13]. In patients using lithium, hypothyroidism and subclinical hypothyroidism have been reported at a rate of occurrence that is six times higher than the rate of occurrence in the normal population [4, 13]. Thyrotoxicosis associated with lithium may also be seen but less frequently than goiter and hypothyroidism. Lithium has been reported to increase the titer of the existing thyroid antibodies, disrupt the structure of thyroglobulin, reduce hepatic deiodination and the clearance of free thyroxin, competitively inhibit iodine transport in the thyroid gland, and, in some cases, reverse the increase in iodine uptake [13]. In our patient, the thyroid dysfunction was considered to be ESS. In the present case, lithium use and other conditions associated with lithium toxicity may, together, have led to thyroid dysfunction. The recovery of thyroid functions after treatment of other pathologies supported this conclusion.

## 4. Conclusion

Lithium is an indispensable agent in the treatment of psychiatric disorders because of its efficacy and cost effectiveness; however, side effects can have insidious and serious consequences. The risk-benefit ratio of chronic lithium use must be evaluated in each individual case. Moreover, to avoid toxicity, lithium should be used at the lowest effective dose, serum lithium levels should be closely monitored, and patients should be evaluated for toxicity and side effects. In the literature, there are a limited number of case reports of lithium-induced multiple clinical conditions. The case presented in this paper is important because it required a multidisciplinary approach to address the multiple clinical manifestations. Additionally, clinicians should keep in mind that lithium-related side effects might trigger or exacerbate each other.
